# Off-label drugs in laryngology– what should the doctor and the patient know about such therapies? A consensus statement of the European Laryngological Society

**DOI:** 10.1007/s00405-025-09406-9

**Published:** 2025-05-13

**Authors:** Malgorzata Wierzbicka, Renata Kopczyk, Michal Zabrodsky, Cesare Piazza, Gauthier Desuter, Peter Klussmann, Ahmed Geneid, Frederic Dikkers

**Affiliations:** 1 Research & Development Centre, Regional Specialist Hospital Wroclaw, Wroclaw, Poland; 2 Faculty of Medicine, Wroclaw University of Science and Technology, Wroclaw, Poland; 3 Faculty of Management, Wroclaw University of Science and Technology, Wroclaw, Poland; 4 Department of Otorhinolaryngology and Head and Neck Surgery, First Faculty of Medicine, University Hospital Motol, Charles University, Prague, Czech Republic; 5 Unit of Otorhinolaryngology–Head and Neck Surgery, ASST–Spedali Civili of Brescia, Brescia, Italy; 6 Department of Medical, Surgical and Radiological Sciences and Public Health, University of Brescia, Brescia, Italy; 7 ENT, Head and Neck Surgery, Antwerp University Hospital, Edegem, Belgium; 8 Department of Otorhinolaryngology, Head and Neck Surgery, Medical Faculty, University of Cologne, Cologne, Germany; 9 Department of Otolaryngology and Phoniatrics-Head and Neck Surgery, Helsinki University Hospital, University of Helsinki, Helsinki, Finland; 10 Department of Otorhinolaryngology-Head and Neck Surgery, Amsterdam UMC Location AMC, University of Amsterdam, Amsterdam, The Netherlands

**Keywords:** Cidofovir, Bevacizumab, Gardasil^®^, Hyaluronic acid, Mitomycin, Legislation

## Abstract

**Purpose:**

Off-label drugs are being used in laryngology. Prescribing of a medicinal product is a decision taken within the relationship between a patient and his/her treating health care provider (HCP). The purpose of this article is to discuss the medicolegal aspects of off-label drug use, to provide recommendations for obtaining informed patient consent for off-label treatment and to propose the place and role of scientific societies and specialist boards in shaping good practices in this area. The final aim is to present recommendations concerning off-label usage and propose special clauses in informed patients consent.

**Methods:**

The literature was reviewed regarding off-label applications in laryngology. Practical information on off-label use in various EU countries was collected.

**Results:**

Registration data and pharmacokinetics of cidofovir, bevacizumab, Gardasil^®^, hyaluronic acid and mitomycin are provided. Off-label prescribing is not prohibited by EU law. Informed consent to treatment with an off-label drug exists in all EU countries. The risk that a court will accept liability of a HCP in case of off-label prescribing is higher than in case of on-label prescribing. If a HCP is held liable for the outcome of a medical treatment, the approval by the competent authorities and professional guideline is a strong defense.

**Conclusion:**

A patient’s precise, explicit consent for the procedures including off-label drugs administration is mandatory. The second prerequisite is defining a need for creating based on recommendations by national or international scientific societies.

## Introduction

There is an increasing number and need for the use of pharmaceutical products outside the scope of a Marketing Authorization (MA). The subject of this report is the presentation of off-label drugs in the field of laryngology. The pharmacological properties, established therapeutic indications, and detailed off-label use will be described, along with relevant literature review.

The terms ‘licensed’, ‘unlicensed’, and ‘off-label’, often used in relation to marketing and prescribing medicinal products, may confuse. To market a medicinal product in the European Union (EU) and Member States requires a Marketing Authorization (‘product license’ MA), for specified indications under specified conditions, regulated by the National Medicines and Healthcare products Regulatory Agencies. This MA includes the product’s agreed terms of use (the ‘label’), described in the Summary of Product Characteristics (SmPC). Prescribing a licensed product outside those terms is called ‘off‐label’ prescribing. Products for which no‐one holds an MA are unlicensed. Prescribers can prescribe authorized products according to the conditions described in the SmPC (‘on‐label’) or outside those conditions (‘off‐label’). The glossary of terms is presented in Table 1.

**Table 1:** Definitions and terms included in the study

Term	Definition
Medicinal product	(a) any substance or combination of substances presented as having properties of preventing or treating disease in human beings; or
	(b) any substance or combination of substances that may be used by or administered to human beings with a view to
	(i) restoring, correcting, or modifying a physiological function by exerting a pharmacological, immunological, or metabolic action, or
	(ii) making a medical diagnosis
Marketing authorization (MA)	Permission granted to a Marketing Authorization Holder legally to sell, supply, or export, procure the sale, supply or exportation, or procure the manufacture or assembly for sale, supply or exportation of a specified medicinal product
Authorized medicinal product (‘licensed product’)	A medicinal product, marketed by a specified company, for which there is in force
	(a) a marketing authorization;
	(b) a certificate of registration as a homeopathic medicinal product;
	(c) a traditional herbal registration; or
	(d) an Article 126a authorization (see below)
Summary of Product Characteristics (SmPC)	That part of the Marketing Authorization that contains essential information for the use of a medicine, including pharmacological properties, authorized indications, qualitative and quantitative information on benefits and harms, information for individualized care, and pharmaceutical information
Article 126a authorization	An EU authorization that can be issued to license a product whose use is justified for public health reasons and that has been imported from another Member State in the European Union
Authorized modes of use	The ways of using the medicinal product as specified in the Summary of Product Characteristics
Unauthorized product (‘unlicensed product’)	A medicinal product for human use in respect of which no marketing authorization has been granted by a relevant licensing authority
Label	A notice describing or otherwise relating to the contents of a medicinal product; the agreed terms of the Marketing Authorization granted in respect of a medicinal product and set out in the Summary of Product Characteristics
Off-label prescribing	Prescribing of an authorized product for use in a way that is not described in the Summary of Product Characteristics
HCP	Health Care Professional

European Union legislation does not regulate the way medicinal products are ultimately used in medical practice. The ultimate responsibility for the definition of health policy and the delivery of health services and medical care, including off-label use, lies with the Member States [Article 168 (7) TFEU]. Given the legal issues, there is a clear need to implement the rules for controlling off-label drugs, thus regulating the use of pharmaceutical products outside the scope of a MA [1]. This action can establish a framework for patient monitoring and data collection [2], ensure that the risk-benefit ratio is favourable for the patients, and encourage pharmaceutical companies to expand their MA [2]. Up to now, the prescribing of a medicinal product, on- or off-label, is a decision taken within the relationship between a patient and his/her treating health care provider (HCP). All medical activities take place in the environment of applicable European and national law, which results on the one hand from the need to protect the patient and, on the other hand, to ensure legal security for the doctor.

Some off-label drug application address specific patient needs that cannot be met using commercially available drugs but also may derive certain economic benefits and convenience for the patients [3]. It is important from the point of view of both the development of medicine and medical proficiency to underline that decisions regarding the method of patient therapy require in-depth knowledge. The purpose of this article is to discuss the medicolegal aspects of off-label drug use, to provide recommendations for obtaining informed patient consent for off-label treatment and to propose the place and role of scientific societies and specialist boards in shaping good practices in this area. To achieve a better understanding, the literature was reviewed regarding off-label applications in laryngology. Practical information on off-label use in various countries was collected. The final aim is to present recommendations concerning off-label usage and propose special clauses in informed patients consent.

## Materials and methods

The research extended to off-label drugs in the field of laryngology. It includes the pharmacological properties, established therapeutic indications and the detailed off-label use description. The components underlying the use of off-label medicines (clinical practice, established medical knowledge, with the regulations in force in the individual Member States) were compared. Gaps were identified where legislation does not support the traditional administration of off-label medicines. Points for discussion and formulas were proposed to complement the patient’s consent to off-label drug administration. Recommendation of good practices in ​​off-label drugs in laryngology practice were proposed.

## Results

### Analysis of selected off-label drugs used in laryngeal diseases

Registration data and pharmacokinetics of selected off-label drugs used in ENT are summarized in Table 2 and described below. This summary constitutes an exemplary scope of information important for the doctor and useful for informing the patient about the procedure.

**Table 2:** Off-label drugs in the field of laryngology

Name of the medical product	Cidofovir	Bevacizumab	Gardasil 9	Hyaluronic acid (HA)	Mitomycin C (MMC)
Active substance	Cidofovir	Bevacizumab L01FG01	Human Papillomavirus 9-valent Vaccine (Recombinant, adsorbed)	Hyaluronic acid	Mitomycin C
Qualitative and quantitative composition	[(S)-1-(3-hydroxy-2-phosphonylmethoxypropyl) cytosine], a nucleoside analogue	Avastin rhuMAB-VEGF	L1 protein in the form of virus-like particles produced in yeast cells (Saccharomyces cerevisiae CANADE 3 C-5 (Strain 1895)) by recombinant DNA technology. Adsorbed on amorphous aluminium hydroxyphosphate sulfate adjuvant (0.5 milligrams Al)	Several different HAs exist, with letters A, B, etc.	Several different products exist
Pharmaceutical form	1 ml contains 75 mg of anhydrous cidofovir 1 vial contains sodium, 2.5 mmol/57 mg	Each milliliter contains 25 mg of bevacizumab. Each vial contains 100 mg of bevacizumab in 4 ml and 400 mg in 16 ml, corresponding to concentrations ranging from 1.4 to 16.5 mg/ml	1 dose (0.5 ml) contains approximately: Human Papillomavirus (HPV) Type 6 L1 protein 30 micrograms; HPV type 11 L1 protein 40 micrograms; HPV type 16 L1 protein 60 micrograms; HPV type 18 L1 protein 40 micrograms; HPV type 31 L1 protein 20 micrograms; HPV type 33 L1 protein 20 micrograms; HPV type 45 L1 protein 20 micrograms; HPV type 52 L1 protein 20 micrograms; HPV type 58 L1 protein 20 micrograms; Suspension for injection. Clear liquid with white precipitate.	Juvederm voluma Syringe of 1 ml containing 20 mg of hyaluronic gel, 3 mg of Lidocaine Hydrochloride and 1 ml of a Phosphate buffer pH 7.2 q.s.	Package commercialized as a 2, 10, 20, or 40 mg powder which can be injected as a solution using sodium chloride as dissolving medium
First registration date	23 IV 1997	1 I 2013	2007	3 IX 2019	1974 in gastric and pancreatic cancers, combined with other chemotherapeutic agents
Pharmacodynamic properties Group/Action	Antivirals for systemic use, nucleosides and nucleotides excluding reverse transcriptase inhibitors, ATC code: J05AB12 Antiviral drug, cytidine analogue, active in vitro and in vivo against human cytomegalovirus (HCMV)	Therapeutic subgroup: L01– Antineoplastic and immunomodulating agents Chemical subgroup: L01FG - VEGF/VEGFR (Vascular Endothelial Growth Factor) inhibitors Pharmacological subgroup: L01F - Monoclonal antibodies and antibody drug conjugates Biologic therapy (BRM, immunotherapy) Cytostatic agent–anti-angiogenetic agent Recombinant human anti-VEGF.	HPV vaccines are based on hollow virus-like particles (VLPs) assembled from recombinant HPV coat proteins.	Medical Device Materials Stored between 2 °C and 25 °C	Disrupting base paring of DNA molecules in the G-1 phase. Inhibiting the formation of RNA and protein synthesis, inhibiting the proliferation of fibroblasts Antibiotic isolated from the *Streptomyces caespitosus* It is noncell cycle-specific, and thus has direct cytotoxic effect even after a short exposure;
Pharmacokinetic properties	The major route of elimination is by renal excretion of unchanged drug by a combination of glomerular filtration and tubular secretion	Due to their size, monoclonal antibodies are not renally eliminated under normal physiological conditions. Catabolism or excretion are the primary processes of elimination. The half-life of bevacizumab is estimated to be 20 days (range of 11–50 days). The clearance (CL) of bevacizumab is approximately 0.207 L/day	Mechanism of action: Gardasil 9 is an adjuvanted non-infectious recombinant 9-valent vaccine. It is prepared from the highly purified virus-like particles (VLPs) of the major capsid L1 protein from HPV types (6, 11, 16, 18, 31, 33, 45, 52, 58). It uses the amorphous aluminium hydroxyphosphate sulfate adjuvant. The VLPs cannot infect cells, reproduce or cause disease. The efficacy of L1 VLP vaccines is thought to be mediated by the development of a humoral immune response.	Sterile pyogenes free physiological solution of cross-linked hyaluronic acid of non-animal origin	Large molecular weight of 334 Dalton keeps it for a longer period confined to the peritoneal cavity, the aimed target region; water soluble; rapidly cleared from the systemic circulation; the half-life of MMC C is 17 min after a 30 mg intravenous bolus administration
Established therapeutic indications	Treatment of cytomegalovirus (CMV) retinitis in adults with acquired immunodeficiency syndrome (AIDS) without renal impairment	In combination with different chemotherapeutical agents-based chemotherapy indicated for treatment of adult patients with metastatic carcinoma: 2004 colon, 2006 lung, kidney and brain, 2009 glioblastoma cancers, ovarian cancer, persistent, recurrent, or metastatic cervical cancer, first-line non-squamous non-small cell lung cancer, metastatic colorectal cancer	Gardasil 9 is indicated for active immunisation of individuals from the age of 9 years against the following HPV diseases: Premalignant lesions and cancers affecting the cervix, vulva, vagina and anus caused by vaccine HPV types. Genital warts (Condyloma acuminata) caused by specific HPV types.	Performance Report divides hyaluronic acid into three major categories: 1 Muscle/skeletal applications, 2 Dermal, facial and eye applications, 3 Adhesion barrier and bulking agent applications	Topically: In ocular and lacrimal duct surgery as a hemostatic agent and as an anti-fibrotic agent. In the upper aerodigestive tract, MMC is mostly used for the prevention of scarring.
Posology and method of administration	Intravenous Serum creatinine and urine protein levels should be taken before each administration	Injection	Suspension for injection.	Maximum 1 ml	Topically
Dosage	Initial treatment: 5 mg/kg; the drug is administered once a week for 2 consecutive weeks. Maintenance treatment: begins 2 weeks after completing the initial treatment, the recommended dose is 5 mg/kg body weight; the drug is administered once every 2 weeks.	Ranged from 25 to 100 mg, median 12,5 mg/0,5 ml, at 3–6-week intervals.	Individuals 9 to and including 14 years of age at time of first injection Gardasil 9 can be administered according to a 2-dose (0, 6–12 months) schedule (see Sect. 5.1). The second dose should be administered between 5 and 13 months after the first dose. If the second 2 vaccine dose is administered earlier than 5 months after the first dose, a third dose should always be administered. Gardasil 9 can be administered according to a 3-dose (0, 2, 6 months) schedule. The second dose should be administered at least one month after the first dose and the third dose should be administered at least 3 months after the second dose. All three doses should be given within a 1-year period. Individuals 15 years of age and older at time of first injection Gardasil 9 should be administered according to a 3-dose (0, 2, 6 months) schedule. The second dose should be administered at least one month after the first dose and the third dose should be administered at least 3 months after the second dose. All three doses should be given within a 1-year period. The use of Gardasil 9 should be in accordance with official recommendations. It is recommended that individuals who receive a first dose of Gardasil 9 complete the vaccination course with Gardasil 9	20 mg/1 ml	Concentrations ranging from 0.1 to 10 mg/ml, most commonly 0,4 mg/ml.
Important treatment considerations	None	Elderly or pediatric population Hepatic, renal insufficiency	Based on epidemiology studies, Gardasil 9 is anticipated to protect against the HPV types that cause approximately: 90% of cervical cancers, more than 95% of adenocarcinoma in situ (AIS), 75–85% of high-grade cervical intraepithelial neoplasia (CIN 2/3), 85–90% of HPV related vulvar cancers, 90–95% of HPV related high-grade vulvar intraepithelial neoplasia (VIN 2/3), 80–85% of HPV related vaginal cancers, 75–85% of HPV related high-grade vaginal intraepithelial neoplasia (VaIN 2/3), 90–95% of HPV related anal cancer, 85–90% of HPV related high-grade anal intraepithelial neoplasia (AIN 2/3), and 90% of genital warts. JoRRP is caused by upper airway infection primarily with HPV types 6 and 11, acquired vertically (mother-to-child) during childbirth. Observational studies in the US and Australia have shown that the introduction of qHPV vaccine since 2006 has led to declines in the incidence of JoRRP at population level.	None	None.
Special warnings and precautions	None	As above	Hypersensitivity to the active substances or to any of the excipients	Standard precautions associated with injectable materials	Post-application irrigation with an isotonic saline solution after the use of MMC seems to render side effects to no systemic toxicity.
Contraindications	Renal insufficiency with creatinine clearance ≤ 55 ml/min or ≥ 2 + proteinuria (≥ 100 mg/dl)]	None	Individuals with hypersensitivity after previous administration of Gardasil 9 or Gardasil/Silgard should not receive Gardasil 9.	None	None
Potential side effects	Neutropenia, rash, headache, iritis, uveitis, hypotony of the eye, nausea, vomiting, diarrhea, pancreatitis, proteinuria, asthenia, chills	Serious adverse reactions in iv administration: Gastrointestinal perforation ranged from 0.3–3%, arterial thromboembolic events (Grade ≥ 3, 5%, surgical complications, including serious and fatal hemorrhage (Grade 3–5) ranged from 0.4–7%, renal injury, Grade 3–4 proteinuria ranged from 0.7–7%, venous thromboembolism (Grade ≥ 3, 11%, hypertension (Grade 3–4, 5–18%)	The most common adverse reactions observed with Gardasil 9 were injection-site adverse reactions (84.8% of vaccinees within 5 days following any vaccination visit) and headache (13.2% of the vaccinees within 15 days following any vaccination visit). These adverse reactions usually were mild or moderate in intensity.	At site of puncture: hematoma, redness, induration, local infection Allergy: type 1 and 4	None reported
Off-label indications in laryngology	Topical, intramucosal in Recurrent Respiratory Papillomatosis (RRP)	Topical, intramucosal in laryngeal Recurrent Respiratory Papillomatosis (RRP)	Recurrent Respiratory Papillomatosis (RRP)	Vocal fold augmentation Passavant ridge augmentation Vocal prosthesis fistula reduction	Prevention of scarring in larynx (anterior commissure) and trachea.
Description of off-label use	HPVs are part of the commensal microflora of human epithelia and some individuals infected with HPV 6 and 11 are unable to eliminate infection and converts that into chronic HPV infection. These findings support the indications for intra-lesional injection of cidofovir directly into the respiratory papilloma’s and if necessary, the whole laryngeal inlet.	HPV viral oncogene products E6 and E7 have been shown to increase vascular endothelial growth factor (VEGF). Increased VEGF provided a potential target for treatment of RRP. Efficacy in patients in severe Juvenile onset RRP (JoRRP) refractory to surgical or other adjuvant medical treatments, significant regression or even complete resolution of laryngeal and tracheal disease in young children	The vaccine may reduce the risk of recurrent HPV-related diseases in individuals previously infected with specific HPV types covered by the vaccine. Gardasil 9 does not serve as a treatment for existing HPV infections or associated conditions.	As above Commercially available Hylan B gel and Restylane	Used topically in concentrations ranging from 0.1 to 10 mg/ml, most commonly 0,4 mg/ml. The number of applications is 1 in most studies. Application times ranged from 2 to 5 min. Many studies report post-application irrigation with an isotonic saline solution after the use of MMC.
Dosage	Doses in topical cidofovir injections: administered concentration of cidofovir– 5 mg/ml, standard given dose– 25 mg, maximum cumulative dosage − 162,5 mg.	Injection dose ranged from 25 to 100 mg, median 12,5 mg/0,5 ml, at 3–6-week intervals	Gardasil 9 can be administered according to a 3-dose (0, 2, 6 months) schedule.	For the volume of injected HA: less than 1 cc or 1 cc of HA was injected in seven (50%) studies. Frequency: Single administration or multiple doses	Concentrations ranging from 0.1 to 10 mg/ml, most commonly 0,4 mg/ml
Expected effect	Antiviral action against HPV 6,11	The inhibition of VEGF to its cell surface receptors leads to a reduction in microvascular growth and spread of RRP	Reduced risk of the need of surgical intervention for RRP	The viscoelastic properties of vocal folds after the injection of HA-based material are similar to the healthy vocal fold in animal studies.	Reduction of scar tissue
Special warnings and precautions	Presumed high level of carcinogenicity; as of 2024 drug import is now allowed only for authorized use for clinical trials.	Standard precautions associated with injectable solution.	None	Hematoma, edema, local hypersensitivity, local inflammation which may be responsible for dysphonia, dyspnea, and dysphagia. The hypothesized mechanisms for adverse reaction include (1) local hypersensitivity to the proteins incidentally produced in the HA manufacturing process, (2) vascular compression or occlusion by the injected HA, and (3) contamination of the injector device.	Post-application irrigation with an isotonic saline solution after the use of MMC leads to no reported side effects.

### Cidofovir

Cidofovir is an antiviral drug approved by the Food and Drug Administration (FDA) for intravenous use to treat cytomegalovirus retinitis in patients with acquired immunodeficiency syndrome. Even though it has not been approved for topical or intralesional use, cidofovir has also been used to treat several cutaneous and mucosal viral lesions. Observations suggest that Human Papilloma Viruses (HPVs) are part of the commensal microflora of human epithelia [4]. Some individuals infected with HPV 6 and 11 are unable to eliminate infection and convert that into chronic HPV infection [4]. These findings support the indications for use of cidofovir in Recurrent Respiratory Papillomatosis (RRP), both children and adults [5–8]. Other antiviral adjunct therapies (acyclovir, ribavirin) have not been proven to be effective in RRP.

The introduction of cidofovir in the treatment of RRP since the late 1990s has resulted in improvements in disease control [8–11]. The indication for adjuvant treatment is high disease activity or spread of lesions beyond the larynx, however the indication is different between caretakers [12, 13]. The use of cidofovir, depending on the study group, ensured remission in approximately 25% of patients, partial remissions in 25%, and no reaction in 50% [14–16] and can lead to a better voice quality [17]. Nevertheless, the effectiveness of cidofovir remains unproven, but the possibility of some therapeutic effects is modestly suggested [6, 18]. The long-term remission rates for patients with RRP are unknown [19].

Side effects of intralesional cidofovir injections include a single case of squamous cell carcinoma arising from squamous papilloma [20], the association between use of cidofovir and dysplastic mucosal changes [21], and the induction of alterations in malignant transformation gene expression [22, 23]. Due to a presumed level of carcinogenicity of cidofovir shown in animal studies, the American RRP Task Force has published guidelines for clinicians administering this medicine [24, 25].There is an ongoing scientific discussion about the dysplastic propensity of topical cidofovir in the human larynx [20, 26, 27]. Single cases of patients which developed dysplasia during intralesional treatment were reviewed [8]. Dysplasia was observed after an average period of 8 months after cidofovir treatment and occurred in 2.7% percentage, which is concurrent with the incidence of spontaneous malignant degeneration of RRP [20]. The European Laryngological Society (ELS) initiated a research project on the side effects of cidofovir in RRP patients [28]. No clinical evidence was found for long-term nephrotoxicity, neutropenia, or laryngeal malignancies after the administration of intralesional cidofovir in RRP [28]. The current amount of use of cidofovir is unknown.

### Bevacizumab

Bevacizumab is a human monoclonal antibody that binds to vascular endothelial growth factor (VEGF) and prevents its interaction with VEGF receptors (VEGFRs). HPV viral oncogene products E6 and E7 have been shown to increase VEGF [29]. Increased VEGF provided a potential target for treatment of RRP. Since the 2010s, the VEG-inhibitor bevacizumab has been administered to adults and children with severe RRP disease, either intra- or sub-lesional, or applied as systemic treatment. The inhibition of VEGF to its cell surface receptors leads to a reduction in microvascular growth and spread of RRP. In an experimental porcine model, it was shown that intra-mucosal injections do not induce detrimental vocal fold changes [30].

Efficacy in patients with severe juvenile onset of RRP (JoRRP), refractory to surgical or other adjuvant medical treatments, was shown with significant regression or even complete resolution of laryngeal and tracheal disease, especially in young children [31]. Intralesional application of bevacizumab was first reported in 2009 [32–34][. The beneficial effect of sub-lesional bevacizumab results were reported in a few sets of cases [35–37]. The largest comprehensive review on bevacizumab demonstrated that intralesional treatment with bevacizumab showed a lower efficacy than systemic application; in 62% of patients, the post-bevacizumab surgical interval (mean follow-up, 1.8 months) was extended when compared to the interval before treatment [38]. An international consensus statement on systemic bevacizumab was reported in 2021 [39]. A survey was performed by Delphi method to address key points supporting its use and provide preliminary guidance surrounding the use of this treatment modality [40]. In the biannual Laryngoscope state of the art article concerning the RRP treatment Bevacizumab has shown promise as a systemic treatment for RRP [41].

### Gardasil^®^

Gardasil is a non-infectious recombinant vaccine designed to prevent infections caused by HPV. Originally, Gardasil^®^ prevented against HPV types 6, 11, 16 and 18. Later, Gardasil-9^®^ was developed. This prevents, in addition to the original four types, against HPV types 31, 33, 45, 52, and 58. In the ongoing pursuit of public health, the development of vaccines against HPV has been a crucial step in preventing these infections and reducing the associated risks. The vaccine targets the seven most prevalent high-risk strains, known for their link to cervical, oropharyngeal and other cancers, as well as types 6 and 11, responsible for genital warts and RRP. By incorporating a wider range of HPV types, Gardasil-9^®^ aims to enhance its protective capabilities compared to the prior quadrivalent vaccine. The vaccine works by introducing virus-like particles that stimulate the immune system to produce antibodies, providing robust protection against future HPV infections.

Gardasil^®^ is typically administered in a series of two or three doses, depending on the age of the individual receiving the vaccine. The recommended age for vaccination is between 11 and 12 years, but it can be given as early as 9 years and up to age 45. Efficacy and safety clinical trials have demonstrated Gardasil’s high efficacy in preventing HPV infections and associated cancers. The vaccine has also shown to be safe, with minor side effects, such as pain at the injection site and fever, being mild and temporary.

While Gardasil^®^ is primarily a preventive measure, it has demonstrated some therapeutic potentials. Research indicates that the vaccine can reduce the risk of recurrent HPV-related diseases in individuals previously infected with specific HPV types covered by the vaccine [42]. It is crucial to note, however, that Gardasil^®^ does not serve as a treatment for existing HPV infections or associated conditions. Systematic reviews have shown that the addition of HPV vaccination was associated with an increase in time between surgeries and a reduction in the number of surgical procedures required for RRP [43–45]. In patients with RRP, the immune response against HPV 6 and 11 is very weak or not detectable at all. Vaccination can induce HPV-specific antibodies, which are also secreted via the mucosa. In RRP, these antibodies can be thought to prevent the appearance of new papillomas locally on the mucosa after surgical treatment [46]. It is also possible that the cellular immune response against HPV-L1 is improved by the induction of HPV-specific T cells.

### Hyaluronic acid

Hyaluronic acid (HA, C_14_H_21_NO_11_) is a natural component synthetized by hyaluronanesynthases of many different cells of the human body. It is a polysaccharide that is not linked to any central protein. HA is massively present within the extracellular matrix. HA increases matrix viscosity and tissue hydration, stimulates angiogenesis and eventually has a role in cellular proliferation. HA is massively utilized in esthetic medicine as a tissue filler allowing body shaping.

The main indication for HA within the ENT discipline is very similar as it is utilized as an organ expander/ tissue filler. In case of vocal fold immobility in abduction, injection of HA within the thyro-arytenoid muscle allows a re-shaping of the glottic vault allowing glottic closure and phonation. Likewise, HA injection around an esophago-tracheal fistula in laryngectomies can treat existing air leakage around a voice prosthesis. According to the long experience of HA tissue injection in ophthalmic, orthopedic and esthetic medicine, HA can be considered as acting on the upper airway’s structures.

A 2022 French nationwide retrospective study showed that no severe complications were observed in sixty office-based HA injection within the ENT area (mostly larynx and oral cavity) [47]. HA injections rarely cause side effects. Mild complications such as local swelling, erythema, induration, tenderness, abscess formation, and decreased elasticity of the vocal folds are rare. Side effects can be treated with steroids or antibiotics and vary significantly in duration, from hours to weeks or months. Long-term sequelae without resolution are exceptional.

### Mitomycin

Topical mitomycin (MMC) is being used as an application following surgery to prevent scarring in circular structures. It was originally known as a hemostatic agent in ophthalmology. Its use as an antifibrotic agent to prevent scarring in circular structures is being applied in ophthalmology, otorhinolaryngology and other specialties.

MMC is an antibiotic isolated from the *Streptomyces caespitosus*. MMC has both antimetabolite and antiproliferative properties. It disrupts base paring of DNA molecules in the G-1 phase, the first stage of the cell cycle in preparation of the fourth stage (mitosis). MMC thus inhibits the formation of RNA and protein synthesis and as such proliferation of fibroblasts is inhibited [48]. Also, the induction of apoptosis in the fibroblasts is induced [49]. In addition, MMC blocks angiogenesis [50]. As MMC induces apoptosis, it can potentially be toxic when given as systemic treatment.

MMC is being applied topically in concentrations ranging from 0.1 to 10 mg/ml, most commonly 0.4 mg/ml. An in vitro study showed optimal effectiveness in a concentration of 0.2 mg/ml [51]. The number of applications is one in most studies. Application times ranged from 2 to 5 min. Topical MMC improved surgical outcomes in many otolaryngologic procedures compared to controls [52]. Many studies report post-application irrigation with an isotonic saline solution after its use. If irrigation with saline was performed, no systemic toxicity was reported [51]. Its apparent safeness was replicated in 2021 [52].

Ineffectiveness of the use of MMC as topical adjuvant therapy in the endoscopic surgical management of laryngotracheal stenosis has been reported [53]. However, this was refuted as applying MMC to the wound immediately after incision and dilation is conceptually incorrect: MMC will then be ineffective, as there will be (almost) no fibroblasts [54].

Comprehensive interpretation of data of a PRISMA review was limited due to heterogeneity in primary outcome, procedure type and study quality [52]. Most studies addressing airway pathology concluded that topical MCC was beneficial. However, this conclusion should be interpreted carefully given the heterogeneity of patients and failure to control many confounding factors [52].

### Off-label use based on the existing paragraphs and judgment

Knowledge of the legal basis for off-label use of drugs, as expressed in existing paragraphs and issued judgments, is crucial for its use [Addendum: ACTS]. The list of European law regulations in the field of off-label drugs is included in the framework regulations and does not constitute a detailed one. EU law consists of the founding treaties (primary legislation) and the provisions of instruments enacted by the European institutions by virtue of them (secondary legislation, regulations, directives, etc.).

It has to be emphasized that EU legislation on MA of medicinal products aims to safeguard public health and to protect the free movement of these products. The European Court of Justice indeed confirmed that “off-label prescribing is not prohibited, or even regulated, by EU law” and that “there is no provision which prevents doctors from prescribing a medicinal product for therapeutic indications other than those for which a marketing authorization has been granted” (T-452/14 Laboratories CTRS v Commission, paragraph 79)]. Off-label use is however recognized as a concept by EU pharmaceutical law (recital 2 of Paediatric) Regulation and pharmacovigilance provisions in Directive 2010/84/EU). To summarize problem of off-label use based on the existing paragraphs and judgment, the off-label use is not covered by EU pharmaceutical legislation.

Off-label use is recognized as a concept by EU law (recital 2 of the Paediatric Regulation and pharmacovigilance provisions in Directive 2010/84/EU, amending Directive No. 2001/83/EC as regards pharmacovigilance). It imposes on MA holders a responsibility to provide any information of products’ use which might influence the evaluation of the benefits and risks of the medicinal product concerned. Some of the pharmacovigilance requirements extend to off-label use. For example, Member States and MA holders are required to collect and report information on suspected adverse reactions arising from the use of medicines outside the terms of the marketing authorization.

### Off-label drugs usage in legal issues and clinical practice in individual member states

The presentation of the rules of off-label medicines usage in national laws and clinical practice in individual Member States is summarized in Table 3. The column headings contain the names of the Member States, and the row headings consider legal torts and off-label clinical practices, consistent for each country.

**Table 3:** Proposed flowchart of the main legal issues relating to off-label usage in the member states

	EU	France	Italy	Belgium	Germany	Netherlands	Finland	Poland	Spain	Czech Republic
Principle, that prohibits to market medicinal products without a MA	-	+	-	-	+	-	+	-	+	+
MS may temporarily authorize the distribution of an unauthorized medicinal product in response to the suspected or confirmed spread of pathogenic agents, toxins, chemical agents or nuclear radiation any of which could cause harm	+	+ Article R5121- 76-1 PHC	+	-	+	+	+	+	+ The Royal Decree 1015/2009	+
The application “must remain exceptional in order to preserve the practical effect of the MA procedure”	+	+	+	-	+	+	+	+	+	+
Interpretation of ‘special need’	+	+	+	+	+	+	+	+	+	+
Interpretation of ‘bona fide unsolicited order’	+	+	+	+	+	+	+	+	+	+
Special points for discussion with the patient	NA	no	The patient gives his consent for this prescription (informed consent). It is advisable to document this carefully.	Patient gives his informed Conseco including his financial “out-of-pocket-contribution) Patient rights law of 2002	The standards relevant to clinical trials of the German Medicinal Products Act (AMG) do not apply to Off-Label-Use, However, it requires a careful risk-benefit assessment and compliance with the general information and documentation requirements.	The patient gives his consent for this prescription (informed consent). It is advisable to document this carefully.	The patient is informed of the off-label usage being part of established treatment practice, effects of medication including possible side effects that may be encountered.	Yes, special points are in the dedicated form included in the set of documents submitted to the bioethics committee	Yes, in the case of compassionate or off-label use, it is established that informed consent “shall be required before administration of the medicinal product”; (Article 8(2)).	Yes, the patient’s informed consent is required and must be included in the documentation. Situations where a medicinal product may be used off-label: In the event that an authorized medicinal product with the required therapeutic properties is not distributed or is not available and at the same time this use is sufficiently scientifically justified.
Consent of the bioethics committee	NA	no	yes, in case of compassionate use drugs	No	No	no	No	yes	no	No
National recommendation of good practices in off-label usage	NA	yes. The evaluation and approval of specific off-label uses is provided by an official expert group	Yes, guided or regulated by medical guidelines, based on the scientific literature	Extended use principle. That is: usage is authorized as long as national recommendations exists for other indications	no	yes dictated by medical guidelines, pharmacotherapeutic manuals and scientific literature. Scientific associations of specialists that prescribe off-label frequently must inform their members about these rules and agreements.	Off-label prescribing may be guided or regulated by medical guidelines, based on the scientific literature	no	yes Real Decreto 1015/2009 in all cases of use of medicines under special conditions, the provisions of Law 41/2002, of 14 November, the basic law regulating patient autonomy and rights and obligations regarding clinical information and documentation in all cases of use of medicines under special conditions, the provisions of Law 41/2002, of 14 November, the basic law	yes governed by the exact wording of the Act on Medicinal Products No. 378/2007 Coll., Section 8(4). Regulating patient autonomy and rights and obligations regarding clinical information and documentation

MA - marketing authorization

MS– Member States

NA - not applicable

Legal issues in the individual Member States differ as to the overarching principle that it is prohibited by law to place medicinal products on the market without MA. This principle is not explicitly expressed in the law of the EU, Poland and Italy. The principle that member states may temporarily authorize the distribution of an unauthorized medicinal product in response to the suspected or confirmed spread of pathogens, toxins, chemical agents or nuclear radiation, any of which may cause harm, applies in all EU countries law. The application of the “unique conditions” and that the off-label usage “must remain exceptional in order to preserve the practical effect of the MA procedure” is obligatory in all EU countries. The interpretations of “special need” and “bona fide unsolicited procurement” are also used in all EU countries.

The clinical practice issues. consent of the bioethics committee or national recommendations of good practices in the field of off-label drugs are also placed in Table 3. This comparison highlights the differences in individual countries. There some substantial differences appear concerning the need of acquisition of the bioethics committee´s consent, which is indispensable in Poland and in Italy. The experiences of individual countries and their scientific achievements in the development of National Recommendation of good practices in off-label usage also remain varied. Off-label prescribing dictated by medical guidelines, pharmacotherapeutic manuals and scientific literature has been defined in all European countries except Poland. In the Netherlands, for example, scientific associations of specialists that prescribe off-label frequently must inform their members about these rules and agreements. The evaluation and approval of specific off-label uses provided by an official expert group is obligatory in France.

The need for special points to be discussed with the patient with or without obtaining written consent to treatment with an off-label drug exists in all EU countries. Only in the Netherlands and Finland the consent does not need to be in in written. In all other countries written informed consent is required and must be included in the documentation file. The comparison in Table 3 showed a fairly uniform legal line among the member states, with no legal acts clearly prohibiting off-labelling. In the lower part of the table, where legal standards are supplemented by clinical practices, there is considerable diversity. There is scope for refining standards and unifying norms here. Thus, the points for discussion with the patient are summarized in Table 4.

**Table 4:** Shortened and summarized details on laryngologist’s mandatory informed consent for off-label drug usage in nine different EU countries

	Italy	France	Belgium	Germany	Netherlands	Finland	Poland	Spain	Czech Republic
1. The type of patient’s disease	Yes	Yes	Yes	Yes^#^	Yes	Yes	Yes*	Yes	Yes^#^
2. Typical methods of treating this disease along with the results of its previous treatment and current condition	Yes	Yes	Yes	Yes^#^	Yes	Yes	Yes*	Yes	Yes
3. Description of the intended off-label drug that needs to be administered, the method of its administration and its potential beneficial effects and superiority over current treatment	Yes	Yes	Yes	Yes^#^	Yes	Yes	Yes	Yes	Yes^#^
4. Arguments not to treat the patient with standard methods and registered drugs	Yes	Yes	Yes	Yes^#^	Yes	Yes	Yes*	Yes	Yes
5. Alternatives to the off-label drug	Yes	Yes	Yes	Yes^#^	Yes	Yes	Yes*	Yes	Yes
6. The potential profit from administering the off-label drug	Yes	Yes	Yes	Yes^#^	Yes	Yes	Yes*	Yes	Yes^#^
7. The effect of the off-label drug on the patient’s general health	Yes	Yes	Yes	Yes^#^	Yes	Yes	Yes*	Yes	Yes
8. The potential side effects of the off-label drug: common, rare, minor, serious	Yes	Yes	Yes	Yes^#^	Yes	Yes	Yes*	Yes	Yes^#^
9. Can failure to use an off-label drug affect (negatively) the treatment result	Yes	Yes	Yes	Yes^#^	Yes	Yes	Yes*	Yes	Yes

*Has to be included into the written consent or Bioethics committee consent form, ^#^ A written consent form is mandatory

## Discussion

The article presents three interrelated aspects that are important in the use of off-label drugs.

Firstly, practitioners still have little knowledge of legislative issues, which may raise concerns about using preparations without MA.

A second problem is the gaps and deficiencies in the law in this medical area. Even though the European legislation does not forbid or subject off-label use to specific conditions, the rest of the European and national legal framework concerning the detailed regulations still applies. Not all medical areas are covered by specific legislation and some of them, which are the responsibility of individual Member States, still do not specify the subject of off-label use. Therefore, an overview of European practice and regulations is offered here.

The third issue discussed is support for the process defined as shared decision-making. The use of medicinal products is in the end decided by the doctor and the patient, who can choose to use a medicinal product off- or on-label. This implies that the liability rules, criminal law and the ethical and professional standards remain applicable to prescribers of off-label use. Therefore, physicians must be aware that by prescribing an unapproved drug, they assume full responsibility for any harmful effects to their patients, even if the patients have consented verbally or written. Therefore, a hypothetical ideal solution should be considered. For the patient this would enhance safety. This would also be necessary for the civil, professional and criminal liability of the doctor who acts in accordance with medical knowledge in order to provide assistance to the patient [55, 56]. This solution should cover two issues: formulation of the patient’s consent to off-label use with an appropriate off-label usage clause, and formulation of the recommendation of good practices in ​​off-label usage.

Considering the common practice of using off-label drugs and the simultaneous legal loopholes in the detailed regulations regarding their use, we are proposing to present the patient with points for discussion when obtaining consent to treatment with an appropriate off-label use clause. It is undoubtedly important to inform the patient in detail about the nature of the treatment and, on this basis, obtain consent to the procedure.

A patient’s medical treatment may fail, or a patient may experience adverse effects from the applied medicinal products. If adverse effects occur, the issue of liability arises. Treatment with a medicinal product can always have unexpected or undesired effects: all medicinal products have associated risks of adverse drug reactions. The risk that a court will accept liability of an HCP in case of off-label prescribing is higher than in case of on-label prescribing. However, if the off-label treatment is considered appropriate, the prescriber will not automatically be deemed liable for damages. The main difference between off- and on-label prescribing with regard to liability is the fact that for on-label prescribing the HCP can rely on the evaluation of the product by the competent authorities, while for off-label use the HCP cannot. If an HCP is held liable for the outcome of a medical treatment, the approval by the competent authorities and professional guideline is a strong defence.

To summarize, in the presented paper the gaps were identified where legislation does not support the traditional administration of off-label medicines. Therefore, a hypothetical ideal solution should be considered. This situation would cover off-label issues in the field of patient safety, providing with full information and allowing to choose the best treatment approach, which would at the same time alleviate the issues of civil, professional and criminal liability of the doctor [57, 58]. The points for discussion with the patient proposed by the authors and the clause in the consent to the procedure fulfil these postulates.

The use of off-label drugs must be consistent with medical knowledge, as shown by the authors for the exemplary products. In extreme cases, inappropriate off-label use could lead to complaints about the prescriber’s misconduct and to an inquiry by a disciplinary board or even to criminal charges. For all off-label prescribers, compliance with the principles of good medical practice and adherence to recommendations issued in this field are of key importance. This is where the role of scientific boards with highly specialized experience in the field comes into play and provide the deep and circumstanced knowledge based on the principles of good clinical practice. The recommendations of scientific societies will not replace the law on the use of off-label drugs, but they can provide guidelines for the use of these preparations as part of good medical practices and contribute to redefining the legal framework.

## Conclusion

Prescribing off-label drugs is not regulated by EU legislation but create a framework and legal environment for national regulations, which are not detailed and precise. Thus, two paths were presented that would best cover off-label issues in the scope of patient safety, civil law, professional, and criminal liabilities. The first is a patient’s precise, explicit consent for the procedures including off-label drugs administration, presented in this paper. The second is defining a need for creating based on the principles of good clinical practice recommendations by national or international scientific societies. Ideally, these prerequisites should be combined.

### Addendum: ACTS:

Directive 2001/83/EC of the European Parliament and of the Council of 6 November 2001 on the Community code relating to medicinal products for human use OJ L 311, 28/11/2001 P. 0067–0128.

Consolidated versions of the Treaty on European Union and the Treaty on the Functioning of the European Union - Consolidated version of the Treaty on the Functioning of the European Union - Protocols - Annexes - Declarations annexed to the Final Act of the Intergovernmental Conference which adopted the Treaty of Lisbon, -TFUE OJ C 326, 26/10/2012 P. 0001–0390.

Directive 2010/84/EU of the European Parliament and of the Council of 15 December 2010 amending, as regards pharmacovigilance, Directive 2001/83/EC on the Community code relating to medicinal products for human use Text with EEA relevance OJ L 348, 31.12.2010, p. 74–99.

COMMISSION REGULATION (EC) No 1234/2008 of 24 November 2008 concerning the examination of variations to the terms of marketing authorisations for medicinal products for human use and veterinary medicinal products OJ L 334, 12.12.2008, p. 7–24.

REGULATION (EC) No 1394/2007 OF THE EUROPEAN PARLIAMENT AND OF THE COUNCIL of 13 November 2007 on advanced therapy medicinal products and amending Directive 2001/83/EC and Regulation (EC) No 726/2004.

Regulation (EC) No 726/2004 of the European Parliament and of the Council of 31 March 2004 laying down Community procedures for the authorization and supervision of medicinal products for human and veterinary use and establishing a European Medicines Agency (Text with EEA relevance) OJ L 136, 30/04/2004 P. 0001–0033.

Regulation (Eu) No 536/2014 Of the European Parliament and of the Council Of 16 April 2014 on Clinical Trials on Medicinal Products for Human Use, And Repealing Directive 2001/20/EC OJ L 158.1 27/5/2014.

Directive 2001/83/EC of the European Parliament and of the Council of 6 November 2001 on the Community code relating to medicinal products for human use OJ L 311, 28/11/2001 P. 0067–0128.

#### CASE

C-185/10 Commission v Poland [2012], JGR 2012/14.

## References

[1] Krause JH (2016) Truth, falsity, and fraud: Off-Label drug settlements and the future of the civil false claims act. Food Drug Law J 71(3):401–440 29140064

[2] Degrassat-Theas A, Bocquet F, Sinegre M, Peigne J, Paubel P (2015) The temporary recommendations for use: A dual-purpose regulatory framework for off-label drug use in France. Health Policy 119(11):1399–1405 26455642 10.1016/j.healthpol.2015.09.003

[3] Kircik L, Siegel DM (2023) Clinical and legal considerations in pharmaceutical compounding. J Clin Aesthet Dermatol 16(8 Suppl 1):S23–S28 37636017 PMC10453396

[4] Bonagura VR, Hatam LJ, Rosenthal DW, de Voti JA, Lam F, Steinberg BM, Abramson AL (2010) Recurrent respiratory papillomatosis: a complex defect in immune responsiveness to human papillomavirus-6 and– 11. APMIS 118(6–7):455–470 20553528 10.1111/j.1600-0463.2010.02617.x PMC2909665

[5] Bielecki I, Mniszek J, Cofala M (2009) Intralesional injection of Cidofovir for recurrent respiratory papillomatosis in children. Int J Pediatr Otorhinolaryngol 73(5):681–684 19193450 10.1016/j.ijporl.2009.01.002

[6] Chung BJ, Akst LM, Koltai PJ (2006) 3.5-Year follow-up of intralesional Cidofovir protocol for pediatric recurrent respiratory papillomatosis. Int J Pediatr Otorhinolaryngol 70(11):1911–1917 16919339 10.1016/j.ijporl.2006.06.018

[7] Gallagher TQ, Derkay CS (2009) Pharmacotherapy of recurrent respiratory papillomatosis: an expert opinion. Expert Opin Pharmacother 10(4):645–655 19284366 10.1517/14656560902793530

[8] Dikkers FG (2006) Treatment of recurrent respiratory papillomatosis with microsurgery in combination with intralesional cidofovir–a prospective study. Eur Arch Otorhinolaryngol 263(5):440–443 16328406 10.1007/s00405-005-1013-3

[9] Pransky SM, Magit AE, Kearns DB, Kang DR, Duncan NO (1999) Intralesional Cidofovir for recurrent respiratory papillomatosis in children. Arch Otolaryngol Head Neck Surg 125(10):1143–1148 10522508 10.1001/archotol.125.10.1143

[10] Ablanedo-Terrazas Y, Soda-Merhy A, Hernandez-Palestina M, Ormsby CE, Reyes-Teran G (2012) Intralesional Cidofovir in severe juvenile respiratory papillomatosis. B-ENT 8(3):197–202 23113383

[11] McMurray JS, Connor N, Ford CN (2008) Cidofovir efficacy in recurrent respiratory papillomatosis: a randomized, double-blind, placebo-controlled study. Ann Otol Rhinol Laryngol 117(7):477–483 18700421 10.1177/000348940811700702

[12] Chadha NK, James A (2012) Adjuvant antiviral therapy for recurrent respiratory papillomatosis. Cochrane Database Syst Rev 12(12):CD005053 23235619 10.1002/14651858.CD005053.pub4 PMC7208975

[13] Best SR, Bock JM, Fowler NB, Raabe EH, Klein AM, Laetsch TW, McClellan K, Rinkel R, Saba NF, Sidell DR et al (2024) A consensus statement on the administration of systemic bevacizumab in patients with recurrent respiratory papillomatosis. Laryngoscope 10.1002/lary.31670 39096091

[14] Naiman AN, Ayari S, Nicollas R, Landry G, Colombeau B, Froehlich P (2006) Intermediate-term and long-term results after treatment by Cidofovir and excision in juvenile laryngeal papillomatosis. Ann Otol Rhinol Laryngol 115(9):667–672 17044537 10.1177/000348940611500903

[15] Chhetri DK, Shapiro NL (2003) A scheduled protocol for the treatment of juvenile recurrent respiratory papillomatosis with intralesional Cidofovir. Arch Otolaryngol Head Neck Surg 129(10):1081–1085 14568791 10.1001/archotol.129.10.1081

[16] Peyton Shirley W, Wiatrak B (2004) Is Cidofovir a useful adjunctive therapy for recurrent respiratory papillomatosis in children? Int J Pediatr Otorhinolaryngol 68(4):413–418 15013606 10.1016/j.ijporl.2003.11.007

[17] Wierzbicka M, Jackowska J, Bartochowska A, Jozefiak A, Szyfter W, Kedzia W (2011) Effectiveness of Cidofovir intralesional treatment in recurrent respiratory papillomatosis. Eur Arch Otorhinolaryngol 268(9):1305–1311 21519834 10.1007/s00405-011-1599-6 PMC3149670

[18] Pamonag MZ, Seery AM, Omari AIA, Alnouri G, Sataloff RT (2023) Intralesional cidofovir: a systematic review of administration protocols and long-term recurrence rates in adult and juvenile recurrent respiratory papillomatosis. J Voice 10.1016/j.jvoice.2023.07.017 37620175

[19] Bielamowicz S, Villagomez V, Stager SV, Wilson WR (2002) Intralesional Cidofovir therapy for laryngeal papilloma in an adult cohort. Laryngoscope 112(4):696–699 12150526 10.1097/00005537-200204000-00019

[20] Broekema FI, Dikkers FG (2008) Side-effects of Cidofovir in the treatment of recurrent respiratory papillomatosis. Eur Arch Otorhinolaryngol 265(8):871–879 18458927 10.1007/s00405-008-0658-0 PMC2441494

[21] Sajan JA, Kerschner JE, Merati AL, Osipov V, Szabo S, Blumin JH (2010) Prevalence of dysplasia in juvenile-onset recurrent respiratory papillomatosis. Arch Otolaryngol Head Neck Surg 136(1):7–11 20083770 10.1001/archoto.2009.179

[22] Donne AJ, Rothera MP, Homer JJ (2008) Scientific and clinical aspects of the use of Cidofovir in recurrent respiratory papillomatosis. Int J Pediatr Otorhinolaryngol 72(7):939–944 18502519 10.1016/j.ijporl.2008.04.003

[23] Donne AJ, Hampson L, He XT, Rothera MP, Homer JJ, Hampson IN (2007) Effects of Cidofovir on a novel cell-based test system for recurrent respiratory papillomatosis. Head Neck 29(8):741–750 17252592 10.1002/hed.20572

[24] Derkay CS, Volsky PG, Rosen CA, Pransky SM, McMurray JS, Chadha NK, Froehlich P (2013) Current use of intralesional Cidofovir for recurrent respiratory papillomatosis. Laryngoscope 123(3):705–712 23070868 10.1002/lary.23673

[25] Derkay C, Multi-Disciplinary Task Force on Recurrent Respiratory P (2005) Cidofovir for recurrent respiratory papillomatosis (RRP): a re-assessment of risks. Int J Pediatr Otorhinolaryngol 69(11):1465–1467 16174538 10.1016/j.ijporl.2005.08.007

[26] Gupta HT, Robinson RA, Murray RC, Karnell LH, Smith RJ, Hoffman HT (2010) Degrees of dysplasia and the use of Cidofovir in patients with recurrent respiratory papillomatosis. Laryngoscope 120(4):698–702 20205173 10.1002/lary.20785

[27] Lott DG, Krakovitz PR (2009) Squamous cell carcinoma associated with intralesional injection of Cidofovir for recurrent respiratory papillomatosis. Laryngoscope 119(3):567–570 19235765 10.1002/lary.20082

[28] Tjon Pian Gi RE, Ilmarinen T, van den Heuvel ER, Aaltonen LM, Andersen J, Brunings JW, Chirila M, Dietz A, Ferran Vila F, Friedrich G et al (2013) Safety of intralesional Cidofovir in patients with recurrent respiratory papillomatosis: an international retrospective study on 635 RRP patients. Eur Arch Otorhinolaryngol 270(5):1679–1687 23377227 10.1007/s00405-013-2358-7

[29] Rahbar R, Vargas SO, Folkman J, McGill TJ, Healy GB, Tan X, Brown LF (2005) Role of vascular endothelial growth factor-A in recurrent respiratory papillomatosis. Ann Otol Rhinol Laryngol 114(4):289–295 15895784 10.1177/000348940511400407

[30] Ahmed MM, Connor MP, Palazzolo M, Thompson ME, Lospinoso J, O’Connor P, Howard NS, Maturo SC (2017) Effect of high-dose vocal fold injection of Cidofovir and bevacizumab in a Porcine model. Laryngoscope 127(3):671–675 27452286 10.1002/lary.26185

[31] Mohr M, Schliemann C, Biermann C, Schmidt LH, Kessler T, Schmidt J, Wiebe K, Muller KM, Hoffmann TK, Groll AH et al (2014) Rapid response to systemic bevacizumab therapy in recurrent respiratory papillomatosis. Oncol Lett 8(5):1912–1918 25289079 10.3892/ol.2014.2486 PMC4186578

[32] Zeitels SM, Lopez-Guerra G, Burns JA, Lutch M, Friedman AM, Hillman RE (2009) Microlaryngoscopic and office-based injection of bevacizumab (Avastin) to enhance 532-nm pulsed KTP laser treatment of glottal papillomatosis. Ann Otol Rhinol Laryngol Suppl 201:1–13 19845188 10.1177/000348940911800901

[33] Zeitels SM, Barbu AM, Landau-Zemer T, Lopez-Guerra G, Burns JA, Friedman AD, Freeman MW, Halvorsen YD, Hillman RE (2011) Local injection of bevacizumab (Avastin) and angiolytic KTP laser treatment of recurrent respiratory papillomatosis of the vocal folds: a prospective study. Ann Otol Rhinol Laryngol 120(10):627–634 22097147 10.1177/000348941112001001

[34] Rogers DJ, Ojha S, Maurer R, Hartnick CJ (2013) Use of adjuvant intralesional bevacizumab for aggressive respiratory papillomatosis in children. JAMA Otolaryngol Head Neck Surg 139(5):496–501 23681032 10.1001/jamaoto.2013.1810

[35] Zelenik K, Kominek P, Stanikova L, Formanek M (2021) Local bevacizumab treatment of Juvenile-Onset respiratory papillomatosis might induce multiple tracheal pyogenic granulomas. Laryngoscope 131(2):E518–E520 32633817 10.1002/lary.28928 PMC7818179

[36] Enrique OH, Eloy SH, Adrian TP, Perla V (2021) Systemic bevacizumab as adjuvant therapy for recurrent respiratory papillomatosis in children: A series of three pediatric cases and literature review. Am J Otolaryngol 42(5):103126 34175693 10.1016/j.amjoto.2021.103126

[37] Hall SR, Thiriveedi M, Yandrapalli U, Zhang N, Lott DG (2021) Sublesional bevacizumab injection for recurrent respiratory papillomatosis: evaluation of utility in a typical clinical practice. Ann Otol Rhinol Laryngol 130(10):1164–1170 33648353 10.1177/0003489421998215

[38] Pogoda L, Ziylan F, Smeeing DPJ, Dikkers FG, Rinkel R (2022) Bevacizumab as treatment option for recurrent respiratory papillomatosis: a systematic review. Eur Arch Otorhinolaryngol 279(9):4229–4240 35462578 10.1007/s00405-022-07388-6 PMC9363326

[39] Sidell DR, Nassar M, Cotton RT, Zeitels SM, de Alarcon A (2014) High-dose sublesional bevacizumab (avastin) for pediatric recurrent respiratory papillomatosis. Ann Otol Rhinol Laryngol 123(3):214–221 24633948 10.1177/0003489414522977

[40] Sidell DR, Balakrishnan K, Best SR, Zur K, Buckingham J, De Alarcon A, Baroody FM, Bock JM, Boss EF, Bower CM et al (2021) Systemic bevacizumab for treatment of respiratory papillomatosis: international consensus statement. Laryngoscope 131(6):E1941–E1949 33405268 10.1002/lary.29343 PMC9034687

[41] Benedict JJ, Derkay CS (2021) Recurrent respiratory papillomatosis: A 2020 perspective. Laryngoscope Investig Otolaryngol 6(2):340–345 33869767 10.1002/lio2.545 PMC8035938

[42] Husein-ElAhmed H (2020) Could the human papillomavirus vaccine prevent recurrence of ano-genital warts? A systematic review and meta-analysis. Int J STD AIDS 31(7):606–612 32438856 10.1177/0956462420920142

[43] Baumanis MM, Elmaraghy CA (2016) Intersurgical interval increased with use of quadrivalent human papillomavirus vaccine (Gardasil) in a pediatric patient with recurrent respiratory papillomatosis: A case report. Int J Pediatr Otorhinolaryngol 91:166–169 27863633 10.1016/j.ijporl.2016.10.032

[44] Smahelova J, Hamsikova E, Ludvikova V, Vydrova J, Traboulsi J, Vencalek O, Lukes P, Tachezy R (2022) Outcomes after human papillomavirus vaccination in patients with recurrent respiratory papillomatosis: A nonrandomized clinical trial. JAMA Otolaryngol Head Neck Surg 148(7):654–661 35653138 10.1001/jamaoto.2022.1190 PMC9164118

[45] Fancello V, Melis A, Piana AF, Castiglia P, Cossu A, Sotgiu G, Bozzo C, King EV, Meloni F (2015) HPV Type 6 and 18 Coinfection in a Case of Adult-Onset Laryngeal Papillomatosis: Immunization with Gardasil. Case Rep Otolaryngol 2015:916023 10.1155/2015/916023 PMC4691470 26783482

[46] Tjon Pian Gi RE, San Giorgi MR, Pawlita M, Michel A, van Hemel BM, Schuuring EM, van den Heuvel ER, van der Laan BF, Dikkers FG (2016) Immunological response to quadrivalent HPV vaccine in treatment of recurrent respiratory papillomatosis. Eur Arch Otorhinolaryngol 273(10):3231–3236 27188508 10.1007/s00405-016-4085-3 PMC5014878

[47] Woisard V, Alexis M, Crestani S, Gallois Y (2022) Safety of office-based flexible endoscopic procedures of the pharynx and larynx under topical anesthesia. Eur Arch Otorhinolaryngol 279(12):5939–5943 35916924 10.1007/s00405-022-07525-1

[48] Djordjevic B, Kim JH (1968) Different lethal effects of mitomycin C and actinomycin D during the division cycle of HeLa cells. J Cell Biol 38(3):477–482 5664219 10.1083/jcb.38.3.477 PMC2108374

[49] Rahbar R, Jones DT, Nuss RC, Roberson DW, Kenna MA, McGill TJ, Healy GB (2002) The role of mitomycin in the prevention and treatment of Scar formation in the pediatric aerodigestive tract: friend or foe? Arch Otolaryngol Head Neck Surg 128(4):401–406 11926915 10.1001/archotol.128.4.401

[50] Hardillo J, Vanclooster C, Delaere PR (2001) An investigation of airway wound healing using a novel in vivo model. Laryngoscope 111(7):1174–1182 11568538 10.1097/00005537-200107000-00009

[51] Li F, Li P, Cai Z, Liu X, Li L, Zhang H, Li H, He Y, Ye L, Yan X (2022) Establishment of two canine models of benign airway stenosis and the effect of mitomycin C on airway stenosis. Int J Pediatr Otorhinolaryngol 159:111205 35700689 10.1016/j.ijporl.2022.111205

[52] Luu K, Tellez PA, Chadha NK (2022) The effectiveness of mitomycin C in otolaryngology procedures: A systematic review. Clin Otolaryngol 47(1):1–13 34310062 10.1111/coa.13839

[53] Yung KC, Chang J, Courey MS (2020) A randomized controlled trial of adjuvant mitomycin-c in endoscopic surgery for laryngotracheal stenosis. Laryngoscope 130(3):706–711 31022311 10.1002/lary.28025

[54] Dikkers FG (2021) In reference to A randomized controlled trial of adjuvant Mitomycin-C in endoscopic surgery for laryngotracheal stenosis. Laryngoscope 131(1):E205 32598041 10.1002/lary.28834

[55] P. P: [Przeciwdziałanie Off-Label use W Prawie Europejskim. Zarys Wybranych problemów, „studia Prawno-Ekonomiczne] counteracting Off-Label use in European law. Outline of selected problems. Legal Economic Stud 2016(100).

[56] RJ K (2014) [Obrót Detaliczny lekami. Zagadnienia prawne] retail trade in medicines. Legal issues. Wolters Kluwer, In., edn. Warsaw

[57] Caminada RPM, Pasmooij AMG, Stoyanova-Beninska V (2022) Off-label prescription of medicines: what do we know about the legislation in EU member States?? Rare Dis Orphan Drugs J 1(1):5

[58] Study on off (2017) -label use of medicinal products in the European union, NIVEL. In.: Dutch National Institute for Public Health and the Environment, and the European Public Health Alliance

